# Traumatic Brain Injury as a Public Health Issue: Epidemiology, Prognostic Factors and Useful Data from Forensic Practice

**DOI:** 10.3390/healthcare12222266

**Published:** 2024-11-13

**Authors:** Michele Ahmed Antonio Karaboue, Federica Ministeri, Francesco Sessa, Chiara Nannola, Mario Giuseppe Chisari, Giuseppe Cocimano, Lucio Di Mauro, Monica Salerno, Massimiliano Esposito

**Affiliations:** 1 Department of Clinical and Experimental Medicine, University of Foggia, 71122 Foggia, Italy; 2 Department of Medical, Surgical and Advanced Technologies “G.F. Ingrassia”, University of Catania, 95121 Catania, Italy; 3 Department of Translational Medical Sciences, Università degli Studi di Napoli “Federico II”, 80125 Naples, Italy; 4 “Rodolico-San Marco” Hospital, Santa Sofia Street, 87, 95121 Catania, Italy; 5 Department of Mental and Physical Health and Preventive Medicine, University of Campania “Vanvitelli”, 80121 Napoli, Italy; 6 Faculty of Medicine and Surgery, “Kore” University of Enna, 94100 Enna, Italy

**Keywords:** traumatic brain injury, epidemiology, forensic pathology, miRNA, hospitalization

## Abstract

Traumatic brain injury (TBI) represents a major public health problem, being a leading cause of disability and mortality among young people in developed countries. Head trauma occurs across all age groups, each experiencing consistently high rates of mortality and disability. This review aims to present an overview of TBI epidemiology and its socioeconomic impact, alongside data valuable for prevention, clinical management, and research efforts. **Methods:** A narrative review of TBI was performed with a particular focus on forensic pathology and public health. In fact, this review highlighted the economic and epidemiological aspects of TBI, as well as autopsy, histology, immunohistochemistry, and miRNA. **Results:** These data, together with immunohistochemical markers, are crucial for histopathological diagnosis and to determine the timing of injury onset, a fundamental aspect in forensic pathology practice. There is compelling evidence that brain injury biomarkers may enhance predictive models for clinical and prognostic outcomes. By clarifying the cause of death and providing details on survival time after trauma, forensic tools offer valuable information to improve the clinical management of TBI and guide preventive interventions. **Conclusions:** TBI is one of the most common causes of death today, with high costs for health care spending. Knowing the different mechanisms of TBI, reduces health care costs and helps improve prognosis.

## 1. Introduction

Traumatic brain injury (TBI) is a major public health concern, ranking as the leading cause of disability and mortality among young adults in developed countries [1].

According to data from the Centers for Disease Control and Prevention, the USA records approximately 403 emergency department visits and 85 hospital admissions per 100,000 people annually, resulting in significant socioeconomic impact [2]. In Europe, the estimated costs attributable to TBI in 2010 were €33 billion (equivalent to approximately USD 49.7 billion in 2017), with direct costs comprising 41% and indirect costs 59% [3]. Given the huge economic burden of TBI, prevention and treatment strategies from a health-economic perspective are imperative [4].

Although young male victims of high-speed motor vehicle collisions were historically considered the most affected group, recent data indicate that TBI epidemiology is evolving [5]. Additionally, the CDC reports a 34% increase in fall-related TBI cases between 2002 and 2006 [6].

Despite variations in TBI case distribution across age groups, all are characterized by high rates of mortality and disability [7]. Furthermore, older adult TBI patients experience worse outcomes compared to their younger counterparts [7]. Admission to trauma centers has significantly reduced post-injury mortality. In recent years, trauma care has undergone major advancements [8,9]. There has also been an improvement in primary prevention, with greater awareness and advancements in intensive care management [10,11,12]. Understanding the distribution of trauma-related deaths is crucial for improving prevention strategies within the healthcare system. Postmortem examinations play a vital role by stratifying TBI cases based on category, gender, and injury severity, thus enhancing data accuracy for proper evaluation [13]. Furthermore, a significant portion of injury-related deaths and disabilities are preventable through effective interventions [14]. Reviewing evolving injury patterns in TBI patients, along with autopsy findings, causes of death, and prognostic factors influencing survival time, is crucial. This evaluation aids in care management and supports preclinical and translational research, as well as preventive initiatives [13,14,15]. Acosta et al. [15] analysed the cause of death of 900 trauma patients, which was 7.3% of all significant trauma admissions, between January 1985 and December 1995. In the first 24 h of admission, 70% of these patients died of central nervous system injuries, cerebral herniation, atlanto-occipital transection, or brain vascular injury. Central nervous system (CNS) injuries were the most common causes of death in the first hour after admission to the hospital. The origins of death between 24 and 72 h after admission were due to CNS injuries from penetrating or blunt trauma. After 72 h, the first cause of death was attributed to acute inflammatory processes, followed by pulmonary embolism and, finally, brain herniation or cerebral edema, or both [15]. Recent data indicate that TBI-related deaths in U.S. emergency departments total approximately 56,000 annually [16]. When adjusted for population size, TBI-related deaths are lower in the European Union compared to the United States. This difference is partly attributed to the higher rate of firearm-related fatalities in the U.S., where head injuries frequently occur [16]. These disparities may also stem from methodological differences in epidemiological studies and variations in hospital admission policies [16]. The elderly, over 60 years of age, are characterized by the highest mortality rate. A recent meta-analysis highlighted an in-hospital mortality rate of 57% and 75% at 6 months after the traumatic event. In children aged 0–4 years, TBI is most commonly caused by falls, followed by blunt trauma and motor vehicle accidents [16]. Among children aged 5 to 14, TBI is mainly caused by falls, followed by sports or recreational injuries, and motor vehicle accidents [5]. Literature indicates significant differences in TBI incidence and causes across age groups.

This review aims to consolidate and present the latest, most robust evidence on TBI pathophysiology, as well as macroscopic and microscopic forensic findings. It focuses on prognostic factors affecting time to death in fatal TBI cases, while outlining the epidemiology, socioeconomic impact, and data, which are critical for prevention, clinical care, and research.

## 2. Pathophysiology of Traumatic Brain Injury

Brain injury is classified as either primary (mechanically induced), occurring at the moment of trauma, or secondary, developing within an already damaged brain. Primary injury involves localized damage to neurons, axons, glia, and blood vessels, including diffuse axonal injury (DAI) caused by shearing, tearing, and stretching forces [17,18,19]. High severity trauma, both in a single event or repetitive events, can cause blood–brain barrier (BBB) damage and cells and liquid leakage, responsible for extravasation of immune cells, as well as poor regulation of molecules, ions, amino acids and proteins, leading to consistent secondary injury [20,21]. Timing of secondary injury occurrence after the trauma onset is variable and strictly related to neurochemical, metabolic, and cellular changes [22]. Secondary brain damage includes ionic homeostasis and inflammatory mediator imbalance, altered release of neurotransmitters, mitochondrial dysfunction, and lipid peroxidation and membrane degradation, contributing to neuronal necrosis and apoptosis. Consequently, understanding the pathophysiology of traumatic brain injury remains a significant challenge [23].

Primary brain injury triggers several biochemical pathways that lead to secondary cell death and neurodegenerative processes. Such mechanisms occur seconds to minutes after the initial insult and can prolong from days to years after trauma [18,24].

Secondary cellular lesions primarily affect the injury site and surrounding tissues. However, brain damage can later spread to other regions, driven by excitotoxicity, oxidative stress, and inflammation that compromise the blood–brain barrier (BBB) [18,19,24,25,26]. These interconnected and overlapping processes ultimately contribute to increased neurodegeneration [25].

Necrosis and apoptosis, the two primary mechanisms of cell death, engage in a complex network of cellular and biochemical interactions in TBI [27].

Additionally, studies have shown that immature brain cells naturally undergo apoptosis under physiological conditions. Since apoptosis plays a crucial role in brain development, it has been hypothesized that it may overlap with necrosis in TBI-related processes [27].

### 2.1. Excitotoxicity

Excitotoxic mechanisms are critical contributors to cellular damage in various neurological disorders. Brain injury causes elevated extracellular glutamate levels directly resulting from trauma. This occurs due to reduced energy for reuptake, amplified release from increased neuronal membrane depolarization, intracellular accumulation, and leakage from axonal damage [28].

The increase of glutamate concentration in extracellular space is responsible for depolarization of injured neighbouring glial cells or neurons, resulting in tissue damage [29].

The rapid release of glutamate into the interstitial space causes excessive stimulation of glutamate receptors, particularly N-methyl-D-aspartate (NMDA) receptors. This leads to ionic dysregulation, including the accumulation of K^+^ in the extracellular space and the influx of Na^+^ and Ca^2+^ through receptor-gated ion channels. Furthermore, glutamate activation prompts the release of calcium from the endoplasmic reticulum, increasing free intracellular Ca^2+^ concentrations [28,29,30,31].

As a result, excitotoxicity induces a metabolic crisis characterized by energy failure, stemming from the brain’s ineffective attempts to restore ionic homeostasis [28,29,30,31].

Elevated intracellular Ca^2+^ levels activate secondary pathways, including calcium-dependent proteases, like calpains and caspases. This cascade also triggers the production of reactive oxygen and nitrogen species, along with mitochondrial damage, ultimately leading to apoptotic processes [28,29,30,31].

### 2.2. Oxidative Stress and Mitochondrial Dysfunction

Secondary injury in TBI is heavily influenced by reactive oxygen species (ROS). TBI disrupts blood flow, causing cerebral hypoxia or ischemia and reducing oxygen and glucose supply to the brain. Consequently, anaerobic metabolism induces a state of acidosis, activating pH-dependent calcium channels [32,33,34,35,36,37].

Elevated cytoplasmic Ca^2+^ levels in neurons disrupt the mitochondrial electron transport chain, leading to increased production of ROS and reactive nitrogen species (RNS) [32,33,34,35,36,37].

The excessive Ca^2+^ concentration may impair mitochondrial function by generating ROS, resulting in the induction of oxidative stress into the axon. Indeed, oxidative stress can be defined as the alteration of the homeostatic process and the biochemical pathways that regulate the production and the removal of ROS [38,39].

As previously mentioned, axonal injury leads to increased cytoplasmic Ca^2+^ concentrations, mitochondrial impairment, ROS generation, and oxidative stress [38]. Johnson et al. reported that ionic alterations in intracellular and extracellular spaces following axonal damage play a crucial role in both axonal degeneration and dysfunction [40]. Another study suggested that post-traumatic Ca^2+^ influx may alter calcium-ATPase function, as demonstrated in an experimental model of optic nerve stretch injury [41]. Another in vitro study confirmed such evidence by demonstrating increased calcium entry after axon stretch [42]. Buki et al. proposed that the pathophysiology of diffuse axonal injury (DAI)—characterized by local axonal cytoskeletal disruptions, mitochondrial dysfunction, cytochrome-c pathway activation, and caspase enzyme activation—may result from calcium influx caused by axolemma perturbations [43].

The formation of the mitochondrial permeability transition pore (mPTP) is implicated in oxidative stress [44]. This internal membrane protein regulates mitochondrial influx and efflux [45,46].

### 2.3. Brain–Blood Barrier Breakdown

In TBI, tensile and compressive forces arise in the brain from direct or indirect acceleration or deceleration impacts. The brain–blood barrier (BBB), due to its elastic nature, is among the first structures to be affected and damaged by trauma [47]. The breakdown of the BBB following experimental brain injury typically follows a biphasic process [20]. Firstly, an increase in BBB permeability can be observed, reaching a maximum within a few hours and subsequently declining. Secondly, from 3 to 7 days following injury, several mechanisms are activated as a brain response to the injury [20]. Key factors contributing to BBB breakdown after trauma include molecules, such as glutamate, ROS, proinflammatory cytokines, and vascular endothelial growth factor A (VEGFA) [20,48]. After injury, an increased glutamate release can be observed from different cells and invading neutrophils. This mechanism increases BBB permeability, induces apoptosis in brain endothelial cells, and enhances ROS production. ROS activation and increased BBB permeability facilitate the post-traumatic invasion of inflammatory cells and cytokines. This process upregulates the expression of endothelial cell adhesion molecules, such as intercellular adhesion molecule-1 (ICAM1) [20,48]. Matrix metalloproteinases (MMPs) are produced by various cells and can be released from inflammatory cells. MMP production disrupts BBB integrity by degrading basal lamina proteins and tight junctions, which increases TNF-α and IL-1β activity, further enhancing BBB permeability and exacerbating post-traumatic neuroinflammation [47,49].

Obviously TBI produces cerebral edema, with increased intracranial pressure, and permanent brain damage and death. The pathogenesis of traumatic brain edema remains unclear, limiting the development of effective therapeutic options [50]. AQP4 has been shown to play a role in cerebral edema formation, but the impact of changes in AQP4 expression under these conditions remains unclear [50].

### 2.4. Neuroinflammation

Despite extensive research on post-traumatic neuroinflammation, understanding the origin of the neuroinflammatory cascade remains crucial [47]. Thrombin plays a key role in neuroinflammation by stimulating proinflammatory mediators, including various cytokines and the chemokine CXCL 1 [49]. However, in TBI there is excessive microglial activation with an increase in pro-inflammatory cytokines [51]. Several studies in rodent models of TBI have shown a correlation between the activation of post-traumatic inflammatory cells (neutrophils and monocytes) and the development of brain edema and tissue damage. Neurons are highly vulnerable to neutrophil activity, particularly under conditions of oxygen and glucose deprivation. Inflammatory cells also increase BBB permeability, triggering and propagating inflammation through the activation of proteolytic enzymes, such as neutrophil elastase (ELANE) and MMP9 [47,52,53,54].

### 2.5. Glial Components’ Involvement

Astrocytes and microglia engage in complex crosstalk with surrounding cells and the microenvironment, playing a crucial role in TBI pathophysiology. Following TBI, increased inflammatory products (proteases, complement factors, and DAMPs) promote inflammatory cascades, with cellular damage [22,55]. The responses of astrocytes and microglia in experimental TBI studies are closely related to the severity of the trauma [53,56]. Additionally, the interplay between the environment, other cell types, and glial responses contributes to the complex nature of reactive gliosis following TBI [22]. Astrocyte activation and subsequent astrogliosis result in increased intermediate filament expression (vimentin and GFAP) and the regulation of neurotrophic factors, like brain-derived neurotrophic factor (BDNF) [57,58]. Furthermore, astrocytes influence extracellular glutamate concentration by reducing glutamate excitotoxicity to neurons, other cells and the environment [59]. Although astrocytes provide neurotrophic support and guide axonal growth following TBI, prolonged astrogliosis deregulates axon regeneration and inhibits functional recovery.

### 2.6. Neurodegeneration

**Figure 1:**
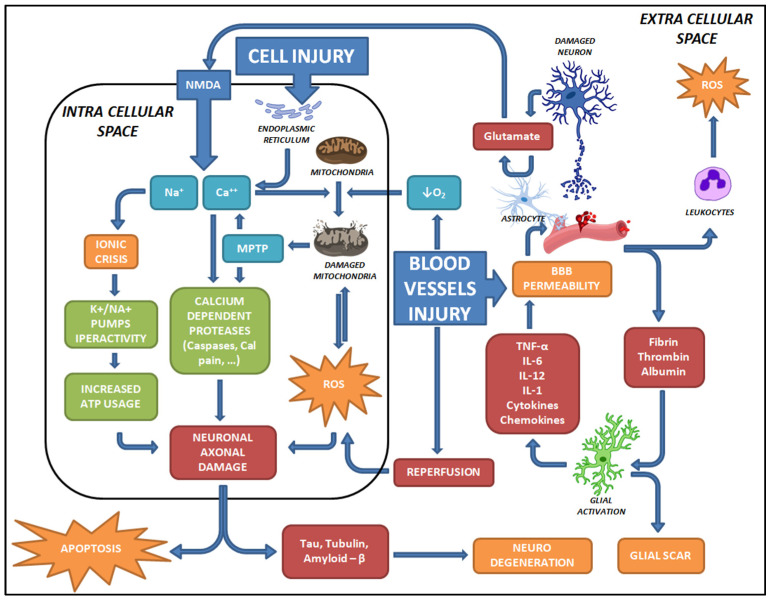
Summary of the metabolic and cascades pathways involved in TBI.

TBI represents a major risk factor for neurodegeneration and cognitive decline in later life. In both human and experimental models, several proteins associated with Alzheimer’s disease (AD) pathophysiology are upregulated. Indeed, the upregulation of β-amyloid precursor protein (APP) may lead to increased production of Aβ [60]. Recent evidence suggests that post-contusion axonal injury and axonal swelling may contribute to long-term sequelae following TBI [8,61,62]. The early phase of TBI is marked by Aβ deposition in the brain and elevated axonal Aβ levels, which are direct consequences of axonal degeneration [63,64,65]. A deeper understanding of the pathological mechanisms underlying TBI is essential for identifying biomolecular markers in forensic histopathology and developing novel therapeutic strategies (Figure 1).

The cell injury, shear or stretching stress, causes the release of intra-cellular Ca^2+^ depots and inability of the endoplasmic reticulum to balance the intra-cellular calcium. Increased calcium concentration causes cell depolarization and the release of glutamate to the extracellular space. Subsequently, NMDA-activated receptors cause neuron depolarization (Excitotoxicity) and Na^+^ and Ca^2+^ influx, which affects ionic balance in the cytosol. The accumulation of Ca^2+^ also activates calcium-dependent proteases, which directly damage cell components and mitochondria. Exposure to elevated Ca^2+^ concentrations leads to mitochondrial dysfunction, causing the release of oxidative radicals and apoptotic factors. These factors damage cellular components through oxidative stress and activate enzymes in the apoptotic pathway. The damaged proteins, as well as structural proteins, are next released in extracellular space, then surviving cells are doomed to neurodegeneration. Injury to blood vessels reduces perfusion and oxygenation, further exacerbating oxidative stress. Reperfusion often coincides with the activation of apoptotic factors, leading to the generation of additional reactive oxygen species. Damage to blood vessels also contributes to the breakdown of the blood–brain barrier, allowing blood-borne proteins (e.g., fibrin, thrombin, albumin) to infiltrate the cerebrospinal fluid. This invasion activates glial cells and elevates the concentrations of pro-inflammatory proteins. Inflammation further weakens the blood–brain barrier and activates leukocytes. These white blood cells contribute to oxidative stress, potentially leading to a chronic neuroinflammatory process through interactions with resident cells.

## 3. Forensic Findings

### 3.1. Macroscopic Findings

There are several classifications of brain injury. Gennarelli et al. proposed a classification of TBI based on the mechanism: contact injuries and acceleration/deceleration injuries (Table 1) [66].

Diffuse brain injury can be divided into diffuse vascular injury, diffuse axonal injury, hypoxic brain injury, and diffuse brain swelling. The nature of the injury depends on the affected brain area and its distribution. Generally, TBI can be classified as focal, including fractures, contusions, subdural hematomas (SDH), or epidural hematomas, or as diffuse, such as diffuse brain edema and diffuse axonal injury [17]. Lesions of the scalp, skull, and dura are crucial in forensic pathology for assessing the site and nature of the underlying injury, as well as for providing information about associated lesions and potential complication [67]. Furthermore, the location and extent of fractures observed during autopsy can offer valuable insights into the trauma dynamics and manner of death [68]. Freeman et al. demonstrated that head injuries in fatal falls are often associated with skull fractures [69]. However, if the body is inverted at the moment of impact, then a basal skull fracture often occurs. The literature emphasizes the importance of a thorough and comprehensive forensic approach when assessing traumatic brain injury, both in living patients and post-mortem, especially in cases involving violent deaths or ill-treatment [70,71]. Six types of contusions have been identified: coup contusions, occurring at the site of impact and causing tensile force injuries to the brain; contrecoup contusions, located on the side opposite the impact point; fracture contusions, associated with skull fractures; intermediate blow contusions, featuring hemorrhagic foci in deep brain structures, like the corpus callosum; sliding contusions, affecting the posterior cerebral lobes; and herniation contusions, resulting from increased intracranial pressure [72].

Extradural hematomas (EDH) are characterized by hemorrhages between the outer dura layer and the inner skull surface. During an autopsy, a scalp contusion is often found on the side of the hematoma, typically accompanied by an underlying fracture and injury to the middle meningeal artery or one of its main branches [73]. Traumatic subdural hematomas can be categorized as acute if symptoms appear within 72 h of trauma, subacute if symptoms develop between 3 days and 3 weeks, or chronic if they manifest more than 3 weeks after the trauma [20]. Subarachnoid hemorrhages are the most frequent result of traumatic head injuries, often occurring alongside surface contusions. Minor subarachnoid hemorrhages are common after trauma and may often be undetectable during an autopsy. One other complicating issue with subarachnoid bleeding is that it frequently occurs as a result of natural diseases, such as vascular malformations. Forensic pathologists face significant challenges in determining whether trauma caused the rupture or if a preexisting rupture led to a fall or other accidents resulting in trauma [68,74]. According to Geddes et al. [75], TBI can be classified into axonal injury (AI), traumatic axonal injury (TAI), and diffuse axonal injury (DAI), with progressively greater axonal damage spreading throughout the brain. Other types of lesions are usually associated with DAI, such as bleeding, gliding contusions, small hemorrhages in the periventricular areas around the third ventricles, as well as intracerebral bleeds in the hippocampus and basal ganglia. In cases of non-formaldehyde-fixed brains, the diagnosis of these injuries is difficult and sometimes missed. Therefore, in such cases, it is always recommended to fix the brain and examine it after autopsy, so as to detect lesions characteristic of DAI [76,77].

### 3.2. Microscopic Findings

Within 18 to 24 h post-injury, damaged axons can be detected using routine hematoxylin and eosin (H and E) staining. After axon injury, damage of adjacent axoplasm occurs, generating the microscopic aspect of retraction bulb [78]. According to a recent study, the term “retraction bulb” is not representative of the pathophysiology of axonal injury, since it is due to dysfunctional axonal transport rather than axonal retraction [79].

Classic microscopic findings include axonal swellings in the cerebral white matter, particularly in patients who died shortly after the injury. In those who died weeks after the injury, small clusters of microglia may be seen throughout the white matter. In those who died months after TBI, Wallerian-like degeneration may be seen in the white matter of the cerebral hemispheres, brainstem, and spinal cord [79].

Li et al. [80] reported a higher number of astrocytes in TBI cases with fatal complications compared to those without. Such data suggest that critical brain injury leads to acute death with no sign of astrocyte activation and that subacute death is associated with progressive brain damage characterized by an astrocyte loss. Furthermore, delayed death cases may be related to astrocyte number. In a human experimental study, Neri et al. [81] demonstrated, in the case of a few days of survival, the presence in the peri-contusional areas of histological signs of edema, such as swelling of astrocytes and dendrites. These lesions were followed by astrocytic swelling, neuronal shrinkage with eosinophilia, nuclear pyknosis, and vasogenic edema. All these lesions appeared in more distant areas, as well as contralateral tissue samples. In the case of higher survival time, an enlargement of astrocytes size occurs with increased soma sizes and thickened processes [81]. To date, β-APP and GFAP are reliable immunohistochemical markers in TBI diagnosis. They have a peculiar localization and pattern distribution after TBI that need to be taken into consideration in TBI diagnosis and timing. Several other markers have been used in forensic pathology to understand the molecular mechanisms involved in brain damage and oxidative stress, such as IL-1β, GFAP, NFL, Spectrin II, 8OHdG, TUNEL, miR- 21, miR-16, and miR-92. Despite difficulties in the use of immunohistochemical markers of early stage TBI due to the overlap of secondary changes after a traumatic event (e.g., hypoxia, edema), which can make survival time estimation difficult and cause errors in estimating survival time, miR-21, miR-92 and miR-16 are reliable biomarkers in postmortem TBI diagnosis as strong predictors of survival [79].

### 3.3. Causes of Death

In-hospital deaths after TBI range from 4.0% to 21.0%, with mortality rates up to 65% higher in patients with comorbid conditions compared to those without [7,82]. Risk factors for in-hospital mortality after TBI are advancing age, race, number of comorbidities, injury severity, and multiple injuries [83]. Complications that occur in the acute care hospital increase the lengths of hospital stays [84]. Death secondary from TBI in the hospital are related to infections, sepsis, venous thromboembolism, and postoperative complications. Specifically, patients with traumatic brain injury could acquire sepsis and respiratory failure more frequently than others [85], and sepsis-associated mortality after injury reaches 37% [7,86]. Recent studies show that traumatic brain injury remains the leading cause of death following multiple trauma, with most fatalities occurring within the first 24 h or during the first week [13,87]. The second most common cause of death is exsanguination, primarily resulting from thoracic or abdominal injuries [88]. Other studies indicate that prehospital mortality accounted for 66% of all TBI deaths, with 27% occurring between 48 and 72 h, 5% between 3 and 7 days, and 2% after 7 days in the hospital [89]. Trunkey et al. described a trimodal distribution of trauma deaths [90]. Immediate deaths from TBI are characterized by severe brain trauma and spinal cord injury. Deaths that occurred within the first few hours after injury and were caused by cerebral hemorrhage. The last group is characterized as late deaths from sepsis or multiorgan failure [91,92]. A recent study indicated a unimodal distribution of road traffic fatalities, showing that most blunt trauma deaths occur at the scene of the accident and may be influenced by alcohol consumption [93,94]. Additionally, the study found that brain injuries, thoracic injuries, and a combination of both were the primary causes of death [95]. According to the previous data, causes of death and mortality patterns in TBI patients and major trauma are essential in establishing the relationship between trauma and death and improving surveillance, prevention and management of such patients. Therefore, autopsy practice should be encouraged in these cases.

### 3.4. Brain Tissue Markers

Numerous biomarkers have been identified for autopsy diagnostics of TBI, marking a critical area of forensic research. The choice of the biomarker to analyze and the site (tissues, biofluids) are dictated by the pathophysiology of TBI. The presence of damage markers and their utility in post-mortem diagnostics are related to their involvement in the pathophysiological mechanisms associated with brain trauma: direct axonal and neuronal damage, cytokine release, neuroinflammation, apoptosis, oxidative stress, altered signal transduction and synaptic plasticity, neurodegeneration, excitotoxicity, and blood–brain barrier disruption [96]. Post-mortem markers of cranial damage encompass both macroscopic and microscopic brain lesions [97]. These markers can provide clues about the nature and extent of the trauma sustained, helping to distinguish between traumatic, ischemic, or degenerative damage. Visual examination of the brain and surrounding structures can reveal contusions, hemorrhages, or cranial deformations. Such evidence is essential for identifying violent traumas, such as those caused by road accidents or assaults. Histology allows examination of brain tissue samples at the cellular level. The presence of apoptosis, necrosis, or infiltration of inflammatory cells can indicate pre-existing or traumatic damage. Specific markers of neuronal distress, detectable through immunostaining, are also utilized. Studies have demonstrated that, following TBI, anti-albumin immunostaining shows positive reactions in frontal cortex neurons, Purkinje cells, and Bergmann glia in the cerebellum [98]. Anti-GFAP immunostaining highlighted the presence of damage at the astrocyte level [99], while suggesting that neuronal immuno-positivity for GFAP is an artifact of processing rather than an actual reactive neuronal presence of GFAP after TBI. Anti-PGRN immunostaining revealed increased expression of the protein in the frontal cortex. Furthermore, anti-CD68 immunostaining showed widespread activation of cortical microglia. These markers are also valuable for determining the timing of TBI events. Following axonal stress, the metabolism of the axon itself varies, which can be recognized by the detection of β-Amyloid Precursor Protein (β-APP). Studies have indicated that, from 0 to 2 h post-mortem, β-APP is weakly visible only in the soma. However, in cases where the survival time was approximately 2 h, damaged axonal foci were found. If the survival time is between 2 to 24 h, there is irregular expression of this marker during this survival period. After 30 days, the expression of β-APP was visible in both gray and white matter; after that, the expression of β-APP became negative again

### 3.5. Biofluid Markers

A fluid biomarker is a measurable molecule in biological fluids, reflecting physiological or pathological processes in the body. The biological fluids analyzed in forensic practice in cases of TBI include cerebrospinal fluid, blood, saliva, urine, and vitreous humor. Following TBI, cell injury leads to the release of cytokines, neuroinflammation, and the activation of glial cells (astrocytes, microglia, and oligodendrocytes) and neurons. The activation of these mechanisms results in the secretion of brain damage markers into circulation. Neuron-specific enolase (NSE) is a key protein in brain tissue, predominantly located in nerve cell bodies and axons. NSE is the only marker that directly evaluates functional damage to neurons. One of the primary issues related to its use as a brain damage marker is hemolysis [96]. Erythrocytes contain high levels of NSE, and hemolysis may, therefore, lead to a significant increase of NSE in the blood [100]. NSE has been detected after 1.5–3 h of survival following trauma [101] and can label damaged axons, but not normal axons, by immunohistochemistry with an intensity comparable to β-APP. Only 20% of cases with a survival time of less than 1.5 h could be identified as TAI (traumatic axonal injury) using the β-APP biomarker, but positive results with NSE were three times higher than those of β-APP [102]. After severe TBI, NSE concentrations in ventricular cerebrospinal fluid are higher in deceased patients than in survivors. However, this marker does not appear to be reliable in serum due to its presence in the walls of red blood cells, which makes the results vulnerable to hemolysis [103]. S100 calcium-binding protein B (S100B) is a well-studied serum biomarker for TBI [104]. Elevated S100B levels have been associated with increased glial activation, progression of secondary injury processes, and poorer prognosis following TBI [105,106]. In serum, S100B was significantly higher in TBI cases compared to other kinds of trauma [107]. Neurofilament light chain (NfL) is considered a more versatile and precise marker of brain injury compared to S100B [108]. However, S100B has several limitations, including a lack of neuronal specificity (its levels can also rise due to physical exertion or polytrauma), a short detection window of three to six hours post-TBI, and variable normal values across different age groups [108]. Glial fibrillary acidic protein (GFAP), an astrocytic biomarker, shows significant increases in cerebrospinal fluid (CSF) and serum levels in cases of TBI fatalities [109]. Interleukin-6 (IL-6) is useful as a TBI marker, particularly in cases of sepsis. The combination of IL-6 and GFAP helps to correctly classify fatal acute TBI in over 90% of cases [110]. Microtubule-associated protein Tau (MAPT) concentration has been observed in biofluids, such as urine and saliva, while no differences are noted in vitreous fluid. Elevated MAPT concentrations in saliva and urine should be considered as a potential marker of both mild and severe TBI in post-mortem examinations. Notably, increased MAPT levels in saliva and urine have been predictive of axonal injury, even in cases where the head trauma was not deemed the direct cause of death, which could be undiagnosed and overlooked during routine forensic autopsies [111].

In conclusion, elevated serum and urine concentrations of progranulin (PGRN) occur during the early phase of TBI [112]. Increased PGRN serum levels have also been observed in patients with acute ischemic stroke [113].

### 3.6. miRNA

miRNAs are short, non-coding RNAs (19–28 nucleotides) crucial for post-transcriptional regulation of gene expression [114]. The central nervous system (CNS) harbors the highest quantity and diversity of miRNAs, with around 70% expressed in the brain, spinal cord, and peripheral nerves [115]. miRNA expression varies during neurological development and across different brain regions [116]. In neurons, miRNAs can localize within specific intracellular compartments, such as axons and dendrites, suggesting that each miRNA may have distinct roles in regulating local protein expression, synaptic maturation, and neural circuit formation. The correct expression of miRNA is essential for the normal development and functioning of the central nervous system (CNS), and alterations in miRNA levels have been associated with cognitive impairments and neuropsychiatric disorders [117]. miRNAs can travel through the extracellular space and reach distant cells, where they influence gene expression, via exosomes and micro-vesicles, or bind to proteins, such as high-density lipoproteins [118]. This protection also allows them to be easily measured in biological fluids, such as serum, plasma, cerebrospinal fluid, urine, and saliva. Thanks to their abundance, stability, and resistance to enzymatic degradation, miRNAs are excellent biomarker candidates [119]. Several studies have investigated alterations in miRNA expression following traumatic brain injury [120]. miR-21 and miR-16 are key biomarkers for TBI, extensively studied in both human and animal models. Both miRNAs are upregulated following brain injury: miR-21 inhibits apoptosis and targets angiogenic factors, helping maintain the blood–brain barrier (BBB). Conversely, miR-16 regulates apoptosis and the cell cycle by targeting molecules like Bcl-2 and cyclin-dependent kinases, promoting neurogenesis and acute repair following TBI. In contrast, some miRNAs, such as miR-107 and miR-27a, undergo downregulation following injury. The loss of miR-107 is a key part of inflammatory processes because it makes granulin possible. Conversely, the loss of miR-27a makes programmed cell death easier by increasing the expression of pro-apoptotic proteins, like Bcl-2. The dynamic regulation of miRNAs highlights their potential as biomarkers for monitoring brain injury. They also hold promise as forensic tools for determining the cause of death in cases of suspected brain trauma [119]. In TBI patients, miRNA 135a and 34b levels increase considerably within the first 24 h following the traumatic event, whereas miR-200c and miR-34c show increased expression after seven days. The dysregulation of miR-34b, miR-135, and miR-451a is closely associated with brain injury and inflammatory processes, suggesting their potential use as biomarkers for damage detection and as prospective therapeutic targets. Conversely, the alteration of miR-34c and miR-200c appears to be involved in neuronal repair processes and the reduction of inflammation, emphasizing their function as indicators in recovery mechanisms [121]. An experimental study published by Sessa et al. [122] analyzed miRNA expression in three groups of cadavers: drug addicts (cocaine users), ischemic stroke victims, and elderly people with age-related damage who died from other neurological causes. miR-132 and miR-34 were upregulated in drug addicts, suggesting a connection to drug-induced neurodegeneration. miR-200b and miR-21 were dysregulated in stroke and age-related cognitive impairment. Additionally, miR-124 was found to be highly sensitive to ischemic damage in individuals who perished from stroke, as evidenced by its increased expression.

Consequently, miRNAs are prospective biomarkers for TBI in the forensic field, as they can assist in the identification of the anatomical region of brain damage, the time of the injury, and the precise cause of death.

## 4. Prognostic Factors and Time of Death

Geographical area, patient age, and injury pattern are crucial factors in determining the time of death and the mortality pattern. Demetriades et al. excluded a specific universal temporal distribution of TBI-related deaths. Indeed, according to these authors, deaths are related to injury mechanisms, injury patterns, injury severity and patient age [123]. Champion et al. conducted a study on trauma patients from 1978 to 2013, finding that prehospital deaths accounted for 56%, and in-hospital deaths for 40% [94].

The Injury Severity Score (ISS) is a reliable medical prognostic score for assessing the severity of trauma, and is related to severity after trauma, specifically mortality, morbidity, and length of hospital stay. If the ISS is greater than 15, a major trauma is defined. Patients with an ISS of 58 ± 2 often die in the pre-hospital setting, while those with an ISS of 28 ± 2 die after 48 h [89].

The Glasgow Coma Scale (GCS) is a widely used tool for assessing mortality and adverse outcomes. However, its reliability is controversial, particularly in populations such as sedated or elderly patients. Another mortality assessment tool is the Abbreviated Injury Score (AIS), which is less influenced by external factors, such as sedation, alcohol, intubation and facial trauma, than the GCS. According to the AIS, the severity of each injury is classified by body region (head, face, neck, chest, abdomen, spine, upper extremities and lower extremities) and by a 1 to 6-point scale. The inter-rater agreement for the degree of severity (therefore similar to the GCS) can be considered a limitation on its usefulness [124].

Despite discrepancies between the Head Abbreviated Injury Score (HAIS) and the GCS, Delhumeau et al. found a strong correlation between the anatomical description of TBI using HAIS and the functional assessment provided by the GCS [125].

## 5. Hospitalization

Observational studies demonstrated a high mortality rate in severe TBI, accounting for 30–40% in observational studies [126]. TBI imposes significant societal costs due to the burden of physical, psychiatric, emotional, and cognitive disabilities among survivors, which also disrupt the lives of patients and their families. TBI is a rising public health concern of considerable proportions. More than 50 million TBIs occur internationally each year [16]. TBI impacts the international economy by approximately USD 400 billion annually, which represents approximately 0.5% of the entire annual global output. Consequently, the economic impact of TBI is substantial [16,127,128,129,130,131,132]. Several models are used to calculate the economic costs of patient and inpatient medical care. Acute hospital care costs have been estimated according to relative value units (RVUs) from Medicare and are based on the relationship between cost-adjusted expenditures, diagnosis, and length of stay [133]. The RVUs are a consequence of the quantity of resources necessary to be used during a hospital stay as a result of a certain diagnosis [134]. The Health Economic Resources Center (HERC) calculates the average daily cost of hospitalization to estimate the overall cost of TBI care [134]. In the United States, the estimated cost of hospitalization for TBI is approximately USD 85.6 billion [135], in Australia, it was estimated at AUD $8.6 billion in 2008. I [16,134]. In Europe, a recent report estimated that yearly costs of TBI accounted for USD 49.7 billion [135,136,137,138], The estimation of higher total costs may be due to the inclusion of intangible costs [16]. Given the significant global economic burden of TBI, it is necessary to enhance prevention and treatment strategies from a health-economic perspective. Besides, there are few data sources regarding costs as a proxy measure of healthcare. Indeed, for both mild and severe TBI, the total cost estimation is still incomplete. This is why it is crucial to collect epidemiological data in order to make an accurate economic estimate and to create a targeted prevention service [16] (Table 2).

## 6. Conclusions

TBI is a major cause of injury-related deaths and hospitalizations worldwide, representing a significant public health concern. Forensic medicine tools, by assessing the cause of death and by giving information regarding the elements involved in time of survival after TBI, are essential in providing useful information for the TBI management field and in promoting preventative efforts. Significant progress has been made in the study of novel clinical variables, blood biomarkers, and neuroimaging in patient characterization aiming to create precision medicine approaches for TBI management. Future studies may address molecular and genomic information in clinical practice. Forensic aspects in TBI are of crucial importance in order to estimate mortality, for example, the use of miRNA as a risk factor for a negative outcome in TBI is being considered. Alternatively, the presence of some biomarkers could, in theory, be used in the future in clinical practice for new scores or added to existing ones, making these more reliable. This would also improve the costs of care and the problems of malpractice claims related to deceased TBI patients after long hospitalization.

## Figures and Tables

**Table 1:** TBI classification according to mechanism of production and neuroradiological and neuropathological findings.

MECHANISM OF PRODUCTION	
Contact	Acceleration/Deceleration
Lesions to scalp Fracture of skull with or without an associated extradural hematoma Surface contusions/lacerations and associated intracerebral hematoma	Tearing of bridging veins with formation of subdural hematoma Diffuse axonal injury including associated intracerebral hematomas Acute vascular injury
NEURORADIOLOGICAL AND NEUROPATHOLOGICAL	
Focal	Diffuse
Injury to scalp Fracture to skull Surface contusions/lacerations Intracranial hematoma Raised intracranial pressure and associated vascular changes	Ischemic damage Axonal injury Brain swelling Meningitis

**Table 2:** Summary of the global cost estimation of TBI and the cost estimation of TBI in different areas according to literature data. All costs have been calculated in USD.

Global Estimation [127,128,129,130,131,132]		
Yearly global traumatic brain injuries estimation	USD 10–50 millions	
Yearly global cost estimation	USD 400 billion	
Based on world production	0.5%	
Mean lifetime cost	USD 222,600 without rehabilitation USD 450,000 post-acute rehabilitation program USD 49,688 annual life care with supervised home placement	
First year healthcare costs	41–53% of lifetime cost (excluded familiar costs)	
European Estimation in 2010 [136]		
Yearly cost estimation	USD 49.7 billion	
Direct costs	41% (USD 20.4 billion)	
Indirect costs	59% (USD 29.3 billion)	
USA Estimation in 2000–2009 [135,137,138]		
	2000	2009
Yearly cost estimation	USD 85.6 billion	USD 252.2 billion
Lifetime medical costs	15%	
Lifetime productivity losses	85%	
Mean lifetime cost	USD 555,424 per patient [137]	
Mean acute cost	USD 33,284–USD 81,153 per patient [138]	
Total direct costs	(USD 81 million)	
Total indirect costs	(USD 2.3 billion)	
Australia Estimation in 2008 [134]		
Yearly costs estimation	USD 7.9 billion	
Productivity losses	55%	
Mean lifetime cost for moderate TBI	USD 124,703 per patient	
Mean lifetime cost for severe TBI	USD 202,456 per patient	

## Data Availability

The authors confirm that the data supporting the findings of this study are available within the article.
